# Plant Core Environmental Stress Response Genes Are Systemically Coordinated during Abiotic Stresses

**DOI:** 10.3390/ijms14047617

**Published:** 2013-04-08

**Authors:** Achim Hahn, Joachim Kilian, Anne Mohrholz, Friederike Ladwig, Florian Peschke, Rebecca Dautel, Klaus Harter, Kenneth W. Berendzen, Dierk Wanke

**Affiliations:** Center for Plant Molecular Biology (ZMBP), Plant Physiology, University of Tübingen, Auf der Morgenstelle 1, Tübingen 72076, Germany; E-Mails: achim.hahn@gmx.net (A.H.); joachim.kilian@uni-tuebingen.de (J.K.); anne.mohrholz@uni-tuebingen.de (A.M.); friederike.ladwig@uni-tuebingen.de (F.L.); florian.peschke@uni-bielefeld.de (F.P.); rebecca.dautel@zmbp.uni-tuebingen.de (R.D.); klaus.Harter@uni-tuebingen.de (K.H.); kenneth.berendzen@uni-tuebingen.de (K.W.B.)

**Keywords:** microarray, type I and type II systemic response, AtGenExpress abiotic stress experiment, plant core environmental stress response (*PCESR*), jasmonic acid, transcriptome

## Abstract

Studying plant stress responses is an important issue in a world threatened by global warming. Unfortunately, comparative analyses are hampered by varying experimental setups. In contrast, the AtGenExpress abiotic stress experiment displays intercomparability. Importantly, six of the nine stresses (wounding, genotoxic, oxidative, UV-B light, osmotic and salt) can be examined for their capacity to generate systemic signals between the shoot and root, which might be essential to regain homeostasis in *Arabidopsis thaliana*. We classified the systemic responses into two groups: genes that are regulated in the non-treated tissue only are defined as type I responsive and, accordingly, genes that react in both tissues are termed type II responsive. Analysis of type I and II systemic responses suggest distinct functionalities, but also significant overlap between different stresses. Comparison with salicylic acid (SA) and methyl-jasmonate (MeJA) responsive genes implies that MeJA is involved in the systemic stress response. Certain genes are predominantly responding in only one of the categories, e.g., *WRKY* genes respond mainly non-systemically. Instead, genes of the plant core environmental stress response (*PCESR*), e.g., *ZAT10*, *ZAT12*, *ERD9* or *MES9*, are part of different response types. Moreover, several *PCESR* genes switch between the categories in a stress-specific manner.

## 1. Introduction

Plants follow a sessile lifestyle and, thus, display a wide ecological plasticity that allows them to adapt to environmental changes by modulating their physiology, growth and development. Although some cells and organs act partly autonomous upon external stimuli, adaptive processes require extensive local and systemic coordination, e.g., during biotic or abiotic stress responses [1–5]. After local stress perception, the information has to be communicated to the rest of the organism by the generation and spread of systemic signals.

Systemic signaling employs various kinds of molecules and is not restricted to stress notification alone. Hormones, such as auxin or strigolactones, are transported from the place of synthesis through the plant to function systemically [6]. In addition, siRNA molecules and possibly ssRNA transmit systemic information and synchronize plant development [7–9]. Some peptides and proteins also constitute mobile signals, such as FT, which is required for flower induction, or TMO7, which is involved in embryonic root specification [10–12].

Salicylic acid (SA) and jasmonate (JA) are well-known systemic signals that are locally induced upon pathogen and herbivore attack and are rapidly transported throughout the plant to established systemic acquired resistance (SAR) in non-challenged tissues [13–16]. In addition, azelaic acid was discovered as a mobile metabolite that most likely primes the plant for SAR and might also activate the secreted, putatively mobile protein AZI1 [17].

Recently, it became evident that reactive oxygen species (ROS) are involved in a multitude of developmental processes and stress response pathways [18–23]. Also electrical signals might contribute as effective systemic signals, which can rapidly be transmitted as action potentials, voltage potentials or as proposed system potentials [24–26]. In addition, a hydraulic signal migrates systemically from the roots to the shoots during drought stress [27,28]. Although the importance of systemic signaling has been known for a long while in plants, it is still not well understood. The characterization of the signaling processes on a genetic basis is difficult, because of the diverse molecule types, which presumably transduce partially redundant systemic information upon various external stimuli.

Studies in yeast have uncovered general stress responses, which were shown to activate similar sets of genes by various stresses [29–31]. These sets of genes are known as common environmental response (CER) in *Saccharomyces cerevisiae*[32] or core environmental stress response (CESR) in *Schizosaccharomyces pombe*[33]. Therefore, CER or CESR can be considered as stereotypical gene expression changes that occur during a multitude of different stresses [30,32,33]. Individual genes, however, might not be necessarily regulated by every stress. It is noteworthy that these stress responses are, at least partly, conserved between *Saccharomyces cereviae* and *Schizosaccharomyces pombe*. While it still remains unknown what kind of roles these genes might play, it seems evident that a common battery of responses can be triggered by various stresses.

In analogy to the common environmental stress responses in yeast, we identified sets of genes that were commonly responsive in several stresses of the AtGenExpress abiotic stress experiment [3,4,34]. A considerable portion of the immediate early responsive genes is differentially expressed in more than only one stress treatment and might represent a plant core environmental stress response (*PCESR*). Importantly, this *PCESR* is not restricted to *Arabidopsis thaliana*, but appears to be conserved between different plant species, such as rice, barley or wheat [3,35–37]. These early-induced, common genes are not related to those responsible for the CESR in yeast cells, which suggests that distinct stress response pathways are required during plant evolution [4,32,33].

Here, we make use of the intercomparability of the nine stresses of the AtGenExpress abiotic stress experiment [4,34]. Six of these stresses were applied to either the shoots or the roots and, hence, allow the comparative investigation of the systemic environmental stress response. We found that gene expression trajectories can be classified into three distinct categories: non-systemic (regulated in the stressed tissue only), systemic type I (regulated in the non-treated tissue only) or systemic type II (regulated in both tissues). Although these categories display distinct functional groups, they still partially overlap between the different stresses and response types. Moreover, meta-analysis suggests that several of the stress responsive gene loci are also SA or JA responsive. We show that type II systemic expression responses are more stress specific than others. In contrast, *PCESR* genes are common to several abiotic stresses, but are rather promiscuous for the response types. Most *PCESR* genes have paralogs that transduce presumably redundant information of the incoming stresses, which reveals a possible backup function.

## 2. Results

### 2.1. Gene Expression Responses Follow a Highly Diverse Pattern during Abiotic Stresses

**Figure 1:**
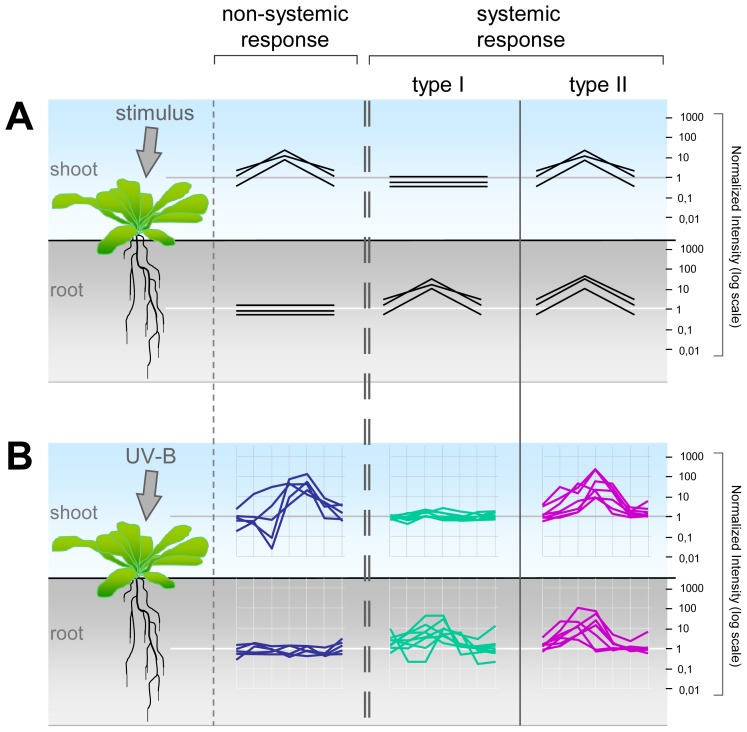
(**A**) Model of the three different gene expression response types in the shoot and root after a stress stimulus; (**B**) Example of non-systemic gene expression trajectories after UV-B irradiation of the shoot, the type I systemic response in the root or the type II systemic response in both tissues. Gene expression changes represent signal intensities normalized to the median and the control experiments.

The analysis of the AtGenExpress abiotic stress experiment disclosed a plethora of gene expression responses also in those tissues that were not directly exposed to the stress treatment [4,34]. We observed both non-systemic and systemic responses to characterize a specific abiotic stress response in *Arabidopsis thaliana*. To dissect these responses and to provide a detailed description of our observations, we need to extend the basic terminology into three principal response types of gene expression (Figure 1). Genes that are differentially expressed in the treated tissue only display a non-systemic response (Figure 1A). We next divided the systemic responses into two categories. A type I systemic response displays gene expression changes exclusively in the non-treated tissue, while, conversely, a type II systemic response is characterized by gene expression changes in both the treated and non-treated tissue (Figure 1A). We exemplified these three principal response types for a subset of genes that were responsive after UV-B light treatment (Figure 1B).

### 2.2. Systemic and Non-Systemic Gene Expression Responses

We applied these definitions to our previously described multidimensional AtGenExpress abiotic stress microarray dataset, which currently provides the unique opportunity of intercomparability between nine different environmental conditions or between the shoot and the root tissue [3,4,34]. Most important for our present analyses are six stresses, *i.e.*, genotoxic, osmotic, oxidative, salt, UV-B and wounding stress, that were applied either to the shoot or the root exclusively. Nevertheless, the three remaining stresses, *i.e.*, drought, cold, and heat, serve as our controls for data validation, because these stresses were simultaneously applied to both tissues and, hence, we can expect overlapping local and systemic responses throughout the entire organism.

To unambiguously categorize differentially expressed genes into the three response types, we applied a chronology-dependency filter to the previously described sets of differentially expressed genes [4]. We require that only those genes are classified, which exhibit differential expression in two consecutive samples with the same trend in both of the replicates. This approach effectively removes outliers and fluctuating gene expression pattern from time series datasets. On the one hand, we are aware that relevant stress-specific information is lost in this process, especially in comparison with the many genes previously identified [4,34]. On the other hand, plain and unequivocal expression response patterns of high informative value are disclosed. These non-systemic, type I and type II systemic responses provide precious information, which are otherwise hidden within complex and partially overlapping expression trajectories.

Indeed, each stress experiment uncovered genes that could be placed in one of the three categories, which indicates the elicitation of systemically activated gene expression after any treatment. In most stress treatments, the highest number of regulated genes is observed locally in the non-systemic organ directly exposed to the stimulus (Table 1; Table S1). In general, the salt, osmotic and UV-B stress treatments affect more genes, compared with genotoxic and wounding stress. There are more systemic type I responsive genes found during the osmotic and oxidative stresses than during the non-systemic response, which underlines a rapid alarm signaling mechanism between the treated and the systemic organs.

Most noteworthy, there was only a single gene of yet unknown function (*At3g20340*) that was type II responsive during oxidative stress (Table 1). In addition, more type I than type II responsive genes are regulated during the osmotic, salt, wounding and oxidative stress treatments.

### 2.3. Functional Categorization Uncovers Specificity in the Different Response Patterns

**Figure 2:**
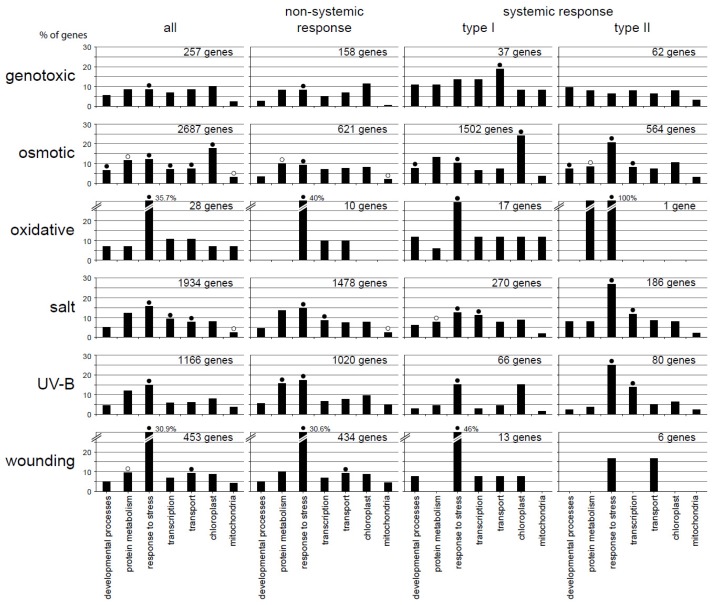
Functional categorization according to the gene ontology (GO) terms. Genes involved in non-systemic, type I systemic or type II systemic responses during the six systemic abiotic stress treatments are categorized for significant over (●)- or under (○)-representation (*p* ≤ 0.01). Percentages of genes categorized into the seven well-annotated GO terms “developmental processes”, “protein metabolism”, “responses to stress”, “transcription”, “transport”, “chloroplast”, or “mitochondria” are given for each of the six stress responses. Number of genes, observed GO counts and *p*-values can be found in Table S2.

A subset of gene ontology (GO) terms was used to classify genes with respect to their putative function (Figure 2). Remarkably, the comparison between all differentially expressed genes and the non-systemic, type I and type II responsive genes revealed significant differences in the GO terms. There is one exception, however, nearly all conditions and categories are enriched for the GO term “stress response,” which is consistent with the types of experiments.

As all of our distinct stress response types contain genes that are linked with “stress response”, we propose that several of those genes are non-specifically responding to many different stress stimuli. This observation can be explained by gene sets that are shared between the GO categories of different treatments, as was proposed before [4], or by genes that switch between our response types in a stress-dependent manner.

Besides the ubiquitous “stress response”, it is noteworthy that some responses involve the categories “developmental processes”, “transcription”, or “organelles”. We noted already that these functional categories are common to almost all stress responses and are shared between different plant species [3]. In addition, significant GO terms differ between the response types and did not correlate with the number of genes [osmotic *vs.* salt *vs.* UV-B] or [genotoxic *vs.* wounding] and, hence, indicate an overall difference in stress response pattern.

### 2.4. Overlap in Non-Systemic, Type I and Type II Systemic Stress Responses

We have described that considerable overlap between stress responses exists [34]. Especially diverse and seemingly unrelated stresses such as cold, drought and UV-B light stress treatments share a set of common genes, in addition to a generally independent transcriptional response pattern [4]. To gain a more detailed insight into the specificity of systemic or local stress responses, we next examined the responsive gene overlap between our non-systemic, type I systemic and type II systemic datasets amongst the six different stresses.

**Figure 3:**
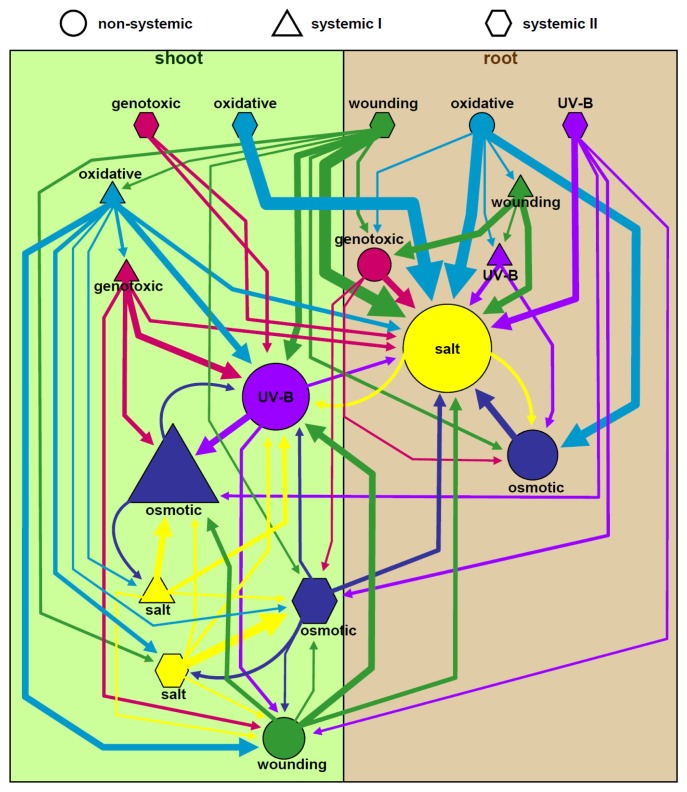
Pair-wise network of gene overlap between the six stresses. Reciprocal comparison of a non-systemic (circle), type I systemic (triangles) or type II systemic (hexagons) responses. The size of each dataset (node) and connecting arrows (edges) represent the overlap of genes shared between two datasets. The sizes of each node or edge are proportional to the amount of overlap (10% to 100%).

A hierarchical graph of the stress responsive gene sets was generated (Figure 3): nodes (gene sets) and directed edges (connecting arrows) are shown proportionally to the total number of genes. As the number of genes inside the sets varies from very few to many regulated genes (Table 1), we choose 10% as the minimal shared rate to equally represent all datasets. The graph separates into two major clusters in the non-systemic expression responses with overlap in the salt and osmotic or the UV-B light and wounding stress responses. This split represents the major differences between the root or the shoot [4].

We can conclude that the principal component of the tissue, to which the stress was applied, dominates over the response type (Figure 3). This tissue-dependent expression is also found in the type I systemic gene sets, even though they are triggered by treatments, which are applied in the non-systemic tissue.

In contrast, the oxidative stress treatment does not activate a unique set of genes and, instead, its genes are found responsive within the other stresses. This is consistent with previous reports about the general involvement of reactive oxygen species (ROS) in many different stresses [18,19,21,34]. Similarly, the genotoxic treatment induces sets of gene expression changes that overlap with many other stresses, but exhibits also specific non-overlapping expression trajectories.

Systemic type II genes have a more complex patterning when compared to the non-systemic or type I systemic gene sets. Interestingly, the type II systemic responses display only little overlap between the type II responses of the other stresses. Instead, significant overlap with the response types of the other stresses exists. Thus, type II systemic response genes are more stress specific than non-systemic or type I systemic genes. In contrast, distinct sets of non-systemic or type I systemic genes respond independently in either the shoot or the root.

### 2.5. The Systemic Stress Response Utilizes Jasmonic Acid

We have shown that the successful stress response requires both the coordinated systemic and non-systemic expression responses. In addition, there is significant overlap between the stress responses in both the treated and the non-treated tissues, which requires yet unknown systemic signaling modules for the rapid coordination of gene expression throughout the entire plant.

**Figure 4:**
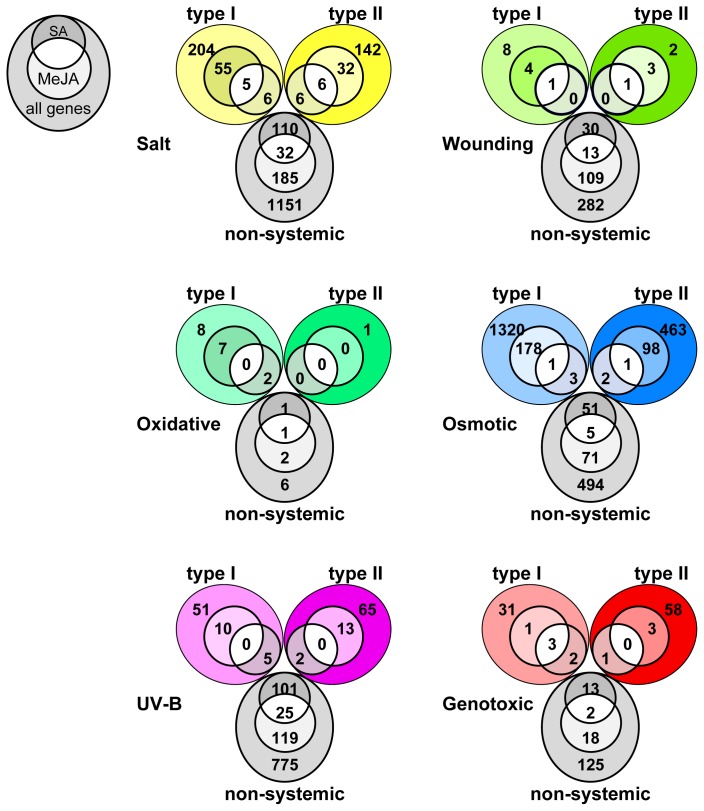
Number of non-systemic, type I systemic or type II systemic genes that respond also to salicylic acid (SA) or methyl-jasmonate (MeJA) treatments.

To address whether already known signaling molecules such as salicylic acid (SA) or methyl-jasmonate (MeJA) might at least partially be involved in the systemic expression responses, we compared our gene lists with already published microarray data on SA and MeJA signaling (Figure 4).

We find SA and MeJA to be involved in the systemic stress responses, as has been described before [14–16,19,38,39]. Interestingly, there is considerable overlap between SA responsive and the non-systemic responsive genes irrespective of the kind of stress. A similar observation is found for MeJA and, thus, both hormones are important for the local non-systemic response. In contrast, type I and type II systemic responses predominantly share genes with the MeJA responses, while the overlap with SA is negligible in the systemic tissue. A portion of genes appears to be under the control of both phytohormones in the non-systemic tissues.

These data suggest that MeJA is a good candidate for the coordination of gene expression in the systemic stress response, whereas the concerted action of SA and MeJA is implicated in the local non-systemic responses.

### 2.6. *WRKY* Genes Are Mainly Involved in the Non-Systemic Stress Response

We have shown that there is a considerable amount of overlap between different stress responses that involves MeJA for its systemic coordination. Gene expression is controlled by transcription factors, and the WRKY transcription factor genes are well known to be responsive during biotic and abiotic stresses [40–43]. To investigate whether these transcription factors might be involved in the coordination of either the type I or type II stress responses, we compared our datasets with all 61 *WRKY* genes present on the microarray. The majority of the *WRKY* genes is not involved in the systemic stress response, but implies a substantial function almost exclusively in the treated non-systemic tissue (Table 2). Consistently, we analyzed the occurrence of *cis*-regulatory elements in each of the response types and found that the cognate W-box binding motif of WRKY transcription factors is exclusively enriched in the non-systemic responsive genes (Table S3).

### 2.7. Plant Core Environmental Stress Response (*PCESR*) Genes

Besides the stress specific gene expression responses, it was noted that a group of genes exists that is differentially expressed in almost any biotic or abiotic stress condition. Therefore, we mined the AtGenExpress abiotic stress experiment for putative Plant Core Environmental Stress Response (*PCESR*) genes that are involved in most of the nine stresses, as was suggested also in previous publications [3,4]. One example of such a gene that meets this definition is *ZAT12*, which is regulated by light, ROS, oxidative, heat, UV-B, cold and drought stress [4,44,45]. Hence, we used *ZAT12*, which encodes a putative zinc-finger transcription factor protein, as a suitable *PCESR* marker.

First, we focused on the overlap between the osmotic, salt and UV-B stress treatments. Only 209 genes are shared between the three stresses, including *ZAT12* (Figure 5).

There are 56 of the 209 genes also differentially expressed after wounding of the leaves. Only five of those 56 are also regulated by genotoxic stress, while four of these are also responsive during the oxidative stress treatment. We, therefore, restrict our set of *PCESR* genes to these 56, as this comprises the best overlap between as many experiments as possible (Figure 5; Table 3). Interestingly, our *PCESR* marker *ZAT12* is not contained in all of the datasets. The reasons are its rapidly changing transcript abundance between the shoot, the root and between the conditions, as well as its temporal expression fluctuations, which has led to its exclusion from the oxidative and UV-B stress dataset. We performed a similar comparison also for the osmotic, salt and UV-B stress treatments and we surprisingly found only nine systemically responsive (Figure S1). This finding also underlines that the systemic stress responses are rather specific to each of the stresses, which we have previously noted (Figure 3).

Most of the previously proposed *PCESR* genes responsive during the seemingly unrelated cold, drought and UV-B light (CDU) stresses are expressed only in the shoots and failed to show up in this analysis due to our stringent filtering criteria (Table 3) [4]. Nevertheless, the vast majority of these CDU responsive genes are also responsive in our systemic datasets examined in this paper and are contained in the 209 genes that overlap between UV-B light, osmotic and salt stress (Figure 5; Table S1) Consistent with the subset of *PCESR* genes identified previously [4], our 56 *PCESR* genes are enriched for genes encoding transcriptional regulators (34%; Table 3). Of the nine genes that are shared between our *PCESR* gene set and the one reported by Kilian *et al.*[4], six are transcription factors, including *ZAT12*. Interestingly, we found nine of our 56 *PCESR* genes to be target gene loci for the H3K27 trimethylation mark that coincides with active repression and silencing of the respective genes in the shoot tissue (Table 3) [46,47]. Instead, we observed a rapid activation of gene expression within a few minutes for several of the presumably silenced *PCESR* genes after application of the stress stimulus. The gene loci of *ZAT12* and *MES9* are rapidly induced in the shoot tissue, although both are also amongst those genes that are H3K27me3 decorated in the shoot. This finding suggests the involvement of rapid chromatin remodeling after the onset of the stress stimulus to mark the *PCESR* genes that are transcriptionally active. During the process of *PCESR* gene identification we found that 30 out of the 56 (54%) genes possess one or more paralogs of presumably redundant function (Table 3) [48]. In the cases of the membrane-associated transcription factors Anac036 and Anac062 [49,50] or the ribonucleases CAF1a and CAF1b [51], the paralogs of the genes are contained in our *PCESR* gene list. This proportionally high number of paralogous *PCESR* genes might be indicative of a putative backup functionality that transduces redundant information of the incoming stresses [48].

### 2.8. *PCESR* Genes Are Systemically Coordinated during Abiotic Stresses

From our observation with *ZAT12*, we have noted that some of the *PCESR* genes might not uniformly follow only one type of expression response or might even be non-responsive under certain conditions. Therefore, our *PCESR* genes are analyzed for their involvement in the non-systemic, type I or type II systemic gene expression responses for each of the six stresses (Table 4).

Intriguingly, the 56 *PCESR* genes exhibit a much more diverse response pattern than what we have previously assumed on the basis of the simple overlap of gene expression responses (Figure 5). In fact, there were only two genes that displayed a coherent response pattern: both the DA1-related gene *DAR3* and the *WRKY18* transcription factor exclusively respond in the non-systemic tissue, which is characteristic at least of the majority of the entire *WRKY* transcription factor family (Table 2). Most of our *PCESR* genes, however, respond in two categories, non-systemically, and in either type I or type II systemic responses, which underlines the probable importance of *PCESR* genes in mediating a specific stress response and to regain plant’s homeostasis. During non-systemic or type I systemic responses, the dirigent gene *DIR5*, which is involved modifying the cell wall, is the only gene that was differentially expressed in all of the six datasets. Interestingly, the two ribonucleases *CAF1a* and *CAF1b* follow predominantly a non-systemic response category. During osmotic stress, however, *CAF1a* displays a type I response, while *CAF1b* is type II responsive.

We recognized that the systemic response pattern is especially prominent during the related osmotic and salt stresses. Almost all of our *PCESR* genes (53/56; 95%) follow a systemic expression response during these two stresses. Moreover, several of the *PCESR* genes switched their response category between these stresses. For example, the heavy-metal-transport superfamily gene *At5g52750* exhibits mainly a type II systemic response, except for the osmotic stress, where it is type I responsive. Likewise, the MATE-transporter *SID1* shows a non-systemic response pattern, while its gene expression is type I responsive during the osmotic stress treatment.

Some *PCESR* genes are well characterized for their involvement in the stress responses in *Arabidopsis*; however, it has not been discovered that their expression response pattern are specifically shifted between the different stress responses: *ZAT10* is also a zinc-finger transcription factor gene like *ZAT12*. However, the expression responses of *ZAT10* are either type I systemic (oxidative stress), type II systemic (osmotic and salt stress) or non-systemic (UV-B light and wounding stress). Likewise, the heavy-metal-transport superfamily gene *At5g52750, ERD9*, methyl-esterase *MES9* or a member of the GNAT-family *At2g32030* exhibit a distinct and specific expression pattern in all three response categories and in a stress-dependent manner. Hence, the majority of *PCESR* genes are likely to respond to various environmental conditions, but with diverse expression trajectories that are stress specific.

### 2.9. Stress-Induced Systemic Signaling Uses Multiple Pathways

We have shown that each of the abiotic stresses did not specifically invoke only a discrete set of genes, but also many genes that are responding specifically to the different stress conditions. We have focused on the analysis of the six abiotic stresses that can be mined for a non-systemic and systemic response pattern. Indeed, most of the genes that respond to the six systemic stresses are also regulated during cold, heat and drought, which were applied to the whole plant and which affected both tissue types (Figure 5). To illustrate that there is considerable overlap of differentially expressed genes between the stresses, we chose to display the three response types for the salt stress response.

The majority of non-systemic as well as type I and type II systemic response genes of the one stress stimulus is also differentially expressed in response to other stresses. Furthermore, systemic responsiveness of a given gene to a certain stress condition did not imply a general role as a systemic response gene (Figure 5), which we have already shown for the *PCESR* genes. Instead, the diverse expression signatures suggest an important, underlying role in homeostatic balance and, thus, an activation by local or systemic signals as needed.

## 3. Discussion

Our detailed analysis of the AtGenExpress abiotic stress data set allowed us to identify genes, which are either non-systemic or of two different types of systemic responses (type I, type II). This provokes the idea of the existence of specific, yet unknown, systemic signaling mechanisms, which must rapidly integrate the stress perception for stress-specific signaling from the treated to the non-treated organ. Based on what is currently known in the literature, the systemic signals could be ssRNA, siRNA, peptides, proteins, JA or electrical signals. The earliest time point in the AtGenExpress dataset is 15 min, which is sufficient time for such a systemic signal to move. Electrical signals take seconds to minutes to be propagated [25]. JA signal transmission (not necessarily JA itself) has been shown to occur within minutes [52–54]. The difficulty in defining stress-specific transcriptional changes is likely due to components that overlap with other stress. For example, mechanical wounding is characterized by the physical rupture of tissues, which affects both ionic fluxes (presumably similar to salt stress) and cellular osmotic potential (osmotic stress treatments). This is in agreement with the observation that wounding elicits responses that overlap with those of biotic and abiotic stresses [55]. Likewise, UV-B treatments activate many genes that are not just specific to UV radiation treatments, but instead had been attributed to pathogen response [56–58], further supporting a connection between abiotic stresses and pathogen defense. The transcriptional wounding response has been shown to overlap with that of JA [15,16,38], which is in turn required for the plant defense against pathogen and herbivore attack [52].

We also observed that genes could be activated either directly or systemically depending on the type of stress, which generates an alarm signal to coordinate responses throughout the entire organism. This has already been proposed in earlier experiments that investigated the systemic activation of transcription by wounding [59], pathogen attack, or the systemic detection of electrical signals [60]. In these publications, it is suggested that an alarm state was raised first followed by the treatment-specific response. Comparable to the systemic leaf-to-leaf signaling described in these publications, in our analysis the establishment of an alarm state is also sent from shoot-to-root or root-to-shoot.

A major outcome of our work is that type II systemic responses appear to be more stress specific than non-systemic or type I systemic responses. This finding might be indicative of the fact that the type II responsive genes have an important function in specifying the stress response, downstream of a mobile systemic signal. In contrast, non-systemic or type I systemic responses displayed considerable overlap also between seemingly unrelated types of stresses or between different stress categories. In fact, Chen *et al.*[33] showed that it was difficult to identify transcriptional responses that could easily be attributed to an individual stress. Similarly, also the intercomparable AtGenExpress abiotic stress experiment suggests the involvement of different molecular pathways that contain the Plant Core Environmental Stress Response (*PCESR*) genes [4].

The *PCESR* genes identified in this work are by no means exhaustive, and as we have shown, many *PCESR* genes will probably be identified from different stress treatments. It is important to note that it is unclear how interlaced the effects on transcription are during different stresses [39]. Nevertheless, the *PCESR* genes identified in this study have several properties that are consistent with a fundamental role in mediating an alarm response. First, they are enriched in transcriptional regulators that are needed for transcriptional re-programming. Second, most of them systemically coordinated and expressed in both the shoot and root. Third, some of the *PCESR* genes are functionally conserved throughout plant evolution, which is a requirement for an effective definition of a plant core environmental stress response, as was proposed for yeast [32,33].

Support that our 56 *PCESR* genes are indeed part of a common alarm system in plants can be taken from the high number of already known genes that have an assigned function in environmental stress responses: *ZAT10* has already been shown to respond to multiple stresses [61,62] and its gene product is known to be involved in diverse developmental pathways. More convincing is the identification of *AtCOR413*-*TM1* and *AtCOR413*-*PM1* amongst the *PCESR* genes. *AtCOR413-TM1* and *-PM1* genes are activated by water stress, ABA, light, freezing tolerance and these responses are conserved between wheat and *Arabidopsis*[36]. An evolutionary conserved function is one of the major arguments that core environmental stress responses exist. AtCOR413-TM1/PM1 are proposed to play a pivotal role in environmental stress signaling and a structural role by stabilizing the plasma membrane lipid bilayer [36]. It is tempting to speculate that gene families evolved and gene duplication occurred to compose a robust system of stress responses. Temperature and UV-B stress have been shown to affect genome stability epigenetically, indicating that abiotic stresses can have heritable effects at least for a few generations [63]. The recent findings that *PCESR* gene expression is increased in Polycomb mutants hints to active repression of some of these genes by H3K27me3 in the non-stressed (shoot) tissue [64]. These findings are supported from a previous publication that describes the dynamic removal of H3K27me3 marks at the *PCESR* gene locus of COR15A during cold stress treatment [65]. Similarly, pathogen attack has been shown to cause an increase in the frequency of somatic DNA recombination [66]. It is, therefore, apparent that responses to stress have short and long-term consequences influencing plant evolution. Not all of our 56 *PCESR* genes responded under all stress stimuli. Especially genotoxic and oxidative stresses revealed little overlap between otherwise commonly involved *PCESR* genes. One explanation is that most of the *PCESR* genes are downstream from a highly specific and well-defined ROS signal [18]. Consistently, the genotoxic and oxidative stresses are presumably affected in ROS production and signaling more generally. A concerted activation of *PCESR* genes under these circumstances might, thus, be impaired.

## 4. Experimental Section

### 4.1. Microarray Data Processing

We used our publicly available AtGenExpress microarray experiment [4], which consists of control arrays (included individually within every stress set at TAIR; 9 time points, 36 chips), cold stress (ME00325, 6 time points; 24 chips), drought stress (ME00338; 7 time points; 28 chips), UV-B light stress, (ME00329; 7 time points; 28 chips), salt stress (ME00328; 6 time points; 24 chips), osmotic stress (ME00327; 6 time points; 24 chips), wounding (ME00330; 7 time points; 28 chips), heat stress (ME00339; without the cell culture data, 8 time points, 32 chips), genotoxic stress (ME00326; 6 time points; 24 chips) and oxidative stress (ME00340; 6 time points; 24 chips).

The arrays were adjusted for the background of optical noise with the GC-RMA package in the R statistical environment of bioconductor [67] and normalized using quantile normalization [4,35,41,68]. Gene expression was normalized to the controls (36 chips) and the 118 conditions (236 chips) present in the entire dataset to identify regulated genes as in Kilian *et al.*[4]. Genes were considered differentially expressed, if they were regulated at least at two consecutive time points (each stress time point was compared to the respective control). Very noisy genes contaminating type II and non-systemic gene sets were removed by hand. The primary normalization was used to generate the principal component analysis. The secondary normalization was used to identify up- or downregulated genes. Centroids were formed for each classification (non-systemic, type I and type II) with k-means set to 2 clusters on the respective tissue. The PCA, regulated genes, and centroids were calculated using GeneSpring GX v7.3.1 (Agilent Technology, Böblingen, German).

### 4.2. Gene List Analysis

A functional categorization using GO-terms was performed with the TAIR web-interface [69]. Significant over- or under-representation (*p* ≤ 0.01) of a particular GO category was assessed by applying a hypergeometric distribution calculation restricted to the universe of all 22,747 genes on the ATH1 array. Calculations were performed in Excel; Results were plotted with plot 0.997 [70]. *Cis*-regulatory element analysis was performed with Athena [71]. Updated using Toronto “_at to AGI converter” 2009-7-29 release [72]. Transcription Factors were found using The Plant Transcription Factor Database (PlnTFDB) [73]. Genes that were hormone responsive were taken from literature [74]. Gene loci that are targeted by histone methylation were derived from publications [46,47].

### 4.3. Network Graph Analysis

The arrays were adjusted for the background of optical noise with the GC-RMA package in the R statistical environment [67,68] and normalized using quantile normalization. Gene expression was normalized to the controls (36 chips) and the 118 conditions (236 Gene lists were compared using the List Distance function from Motif Mapper v5.2.4.01 [75,76]. The values for direct overlap were used as input for Cytoscape v.2.6 [77] and arranged using the yFiles hierarchal clustering algorithm (Table S4). Node size was continually scaled to list size; edge thicknesses were continually scaled to overlap-percentage.

## 5. Conclusions

To conclude, the different stress treatments share an initial alarm state even before the plant can determine exactly what type of stress is actually there. We propose that unknown systemic signaling modules must be responsible for this coordination. However, our categorization of the gene expression responses into the different non-systemic type I and type II systemic stress responses allowed for the first time the in-depth analysis and characterization of six abiotic stresses. Moreover, *PCESR* genes have been identified and have been shown to display a highly stress-specific response pattern. Some of the *PCESR* genes switch between non-systemic, type I and type II systemic responses in a stress-specific manner. In addition, the type II stress responsive genes were found to be more stress specific than the non-systemic or the type I systemic responsive genes. The identification of the mobile signals and the analysis of how a specific response type is enforced at distinct gene loci will be a challenging task for future research.

## Figures and Tables

**Figure 5:**
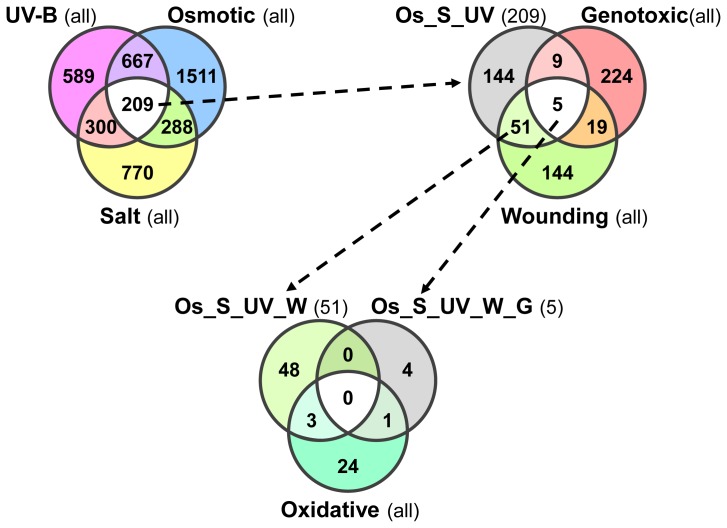
Number of putative *PCESR* genes that responded during most abiotic stress treatments. Overlapping UV-B light, osmotic and salt stress responsive genes are labeled Os_S_UV. Wounding, UV-B light, osmotic and salt stress responsive genes (Os_S_UV_W) or genotoxic, wounding, UV-B light, osmotic and salt stress responsive genes (Os_S_UV_W_G) are indicated accordingly. The base number of genes contained in each set is indicated in parenthesis.

**Figure 5:**
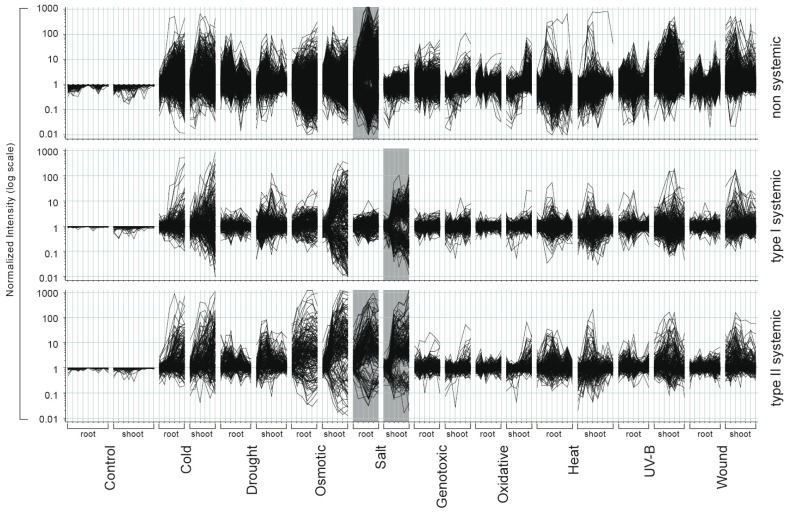
Expression profiles of salt stress response genes. Genes of the non-systemic, type I systemic and type II systemic salt stress response are visualized for all nine treatments of the AtGenExpress abiotic stress experiment. Each line represents the expression trajectory of one gene for each stress condition, in each tissue and along all points in time. The grey bar indicates those genes of the salt stress response that were used as input. Each of the three panels displays individually the non-systemic, systemic type I and systemic type II salt response genes that passed our strict filtering criteria.

**Table 1:** Number of genes involved in a systemic or non-systemic stress response. Genes were classified as regulated under high-stringency conditions, as was described in the text. Identification of significantly regulated genes was conducted for shoot and root datasets independently. Detailed lists of genes are given in Table S1.

	Total	Root	Shoot	Non-Systemic	Systemic Type I	Systemic Type II
Genotoxic	257	220	99	158	37	62
Osmotic	2,687	1,185	2,066	621	1,502	564
Oxidative	28	11	18	10	17	1
Salt	1,934	1,664	456	1,478	270	186
UV-B	1,166	146	1,100	1,020	66	80
Wounding	453	19	440	434	13	6

**Table 2:** Number of *WRKY* genes involved in a systemic or non-systemic abiotic stress response. The 61 *WRKY* genes present on the ATH1 Genechip Array are compared with all genes in each response type.

	Non-Systemic	Systemic Type I	Systemic Type II
Genotoxic	2	1	1
Osmotic	6	4	3
Oxidative	1	0	0
Salt	21	1	0
UV-B	8	0	2
Wounding	4	1	0

**Table 3:** The 56 genes of the plant core environmental stress response (*PCESR*). All genes are regulated during UV-B, osmotic, salt and wounding stress. CDU: genes that were previously proposed as *PCESR* genes [4] during cold, drought and UV-B (CDU) treatment. TR: gene products that are involved in the regulation of transcription (TR). H3K27me3: genes that are known targets for histone methylation in the shoot [46,47]; Paralog: genes with paralogs contained in the list (x), genes that have paralogs in the genome (p), probes that identify paralogous genes (o).

Affy ID	AGI	Description	CDU	TR	H3K27me3	Paralog
263584_AT	At2g17040	Anac036		x		x
252278_AT	At3g49530	Anac062		x		x
257644_AT	At3g25780	Allene oxide cyclase 3				p
264217_AT	At1g60190	AtPUB19 E3 ubiquitin ligase				p
246099_AT	At5g20230	Blue-copper binding SAG14			x	
265480_AT	At2g15970	AtCOR413-PM1/Cyclophilin 19				
259789_AT	At1g29395	AtCOR413-TM1				p
255479_AT	At4g02380	AtLEA5/SAG21				p
246272_AT	At4g37150	Methyl esterase 9			x	p
252053_AT	At3g52400	AtSYP122 syntaxin				
253485_AT	At4g31800	AtWRKY18		x		p
257022_AT	At3g19580	AZF2	x	x		p
252679_AT	At3g44260	CAF1a/CCR4-associated factor 1	x	x	x	x
249928_AT	At5g22250	CAF1b/CCR4-associated factor 1		x		x
250149_AT	At5g14700	Cinnamoyl-CoA reductase-related				
263497_AT	At2g42540	AtCOR15A			x	p
254232_AT	At4g23600	AtCORI3			x	p
247071_AT	At5g66640	AtDAR3				p
252102_AT	At3g50970	Dehydrin XERO2				
252265_AT	At3g49620	Dark inducible 11			x	p
256526_AT	At1g66090	TIR-NBS disease resistance protein	x			
249264_S_AT	At5g41740 At5g41750	TIR-NBS disease resistance proteins				o
262325_AT	At1g64160	Dirigent family protein AtDIR5				
264436_AT	At1g10370	ERD9				p
261470_AT	At1g28370	ERF11	x	x		p
248799_AT	At5g47230	ERF5	x	x		p
265276_AT	At2g28400	Protein of unknown function				
259445_AT	At1g02400	Gibberellin oxidase 6				
266555_AT	At2g46270	G-box binding factor 3		x		
265725_AT	At2g32030	GNAT family protein		x		p
266142_AT	At2g39030	GNAT family protein		x	x	p
251200_AT	At3g63010	Gibberellin receptor GID1B				
258792_AT	At3g04640	RNA-binding glycine-rich protein	x			
250279_AT	At5g13200	GRAM domain-containing protein				
262930_AT	At1g65690	LEA-related/HIN1-related				
246214_AT	At4g36990	Heat shock factor protein 4		x		
248327_AT	At5g52750	Heavy-metal-transport superfamily				p
265724_AT	At2g32100	Ovate family protein 16		x		p
250793_AT	At5g05600	Fe(II)-dependent oxygenase				
248164_AT	At5g54490	EF-hand containing PBP1				
264580_AT	At1g05340	Protein of unknown function				p
256933_AT	At3g22600	Lipid-binding protein				p
252470_AT	At3g46930	Protein kinase family protein				p
258650_AT	At3g09830	Protein kinase family protein				
266834_S_AT	At2g30020 At3g27140 At4g08260	Protein phosphatase 2C				o
251259_AT	At3g62260	Protein phosphatase 2C				
253140_AT	At4g35480	RING finger protein RHA3B		x		p
252921_AT	At4g39030	MATE-transporter SID1/EDS5				
256017_AT	At1g19180	TIFY10A/JAZ1		x		p
254321_AT	At4g22590	Trehalose-6-phosphate phosphatase				
266452_AT	At2g43320	Putative methyltransferase				
250796_AT	At5g05300	Protein of unknown function			x	
254500_AT	At4g20110	Vacuolar sorting receptor 7				p
261648_AT	At1g27730	ZAT10	x	x		
247655_AT	At5g59820	ZAT12	x	x	x	
245711_AT	At5g04340	ZAT6	x	x		

**Table 4:** The 56 *PCESR* genes are involved in different response types. The genes are listed according to a non-systemic (nsy) or type I (I) or type II (II) systemic response. As indicated, several *PCESR* genes do not respond in all of the stresses (●).

AGI	Description	oxidative	genotoxic	osmotic	salt	UV-B	wounding
At5g66640	AtDAR3	•	nsy	nsy	nsy	•	nsy
At4g31800	AtWRKY18	•	•	nsy	nsy	•	nsy
At1g64160	Dirigent family protein AtDIR5	nsy	nsy	nsy	nsy	I	I
At5g05600	Fe(II)-dependent oxygenase	•	•	II	II	nsy	II
At1g27730	ZAT10	I	•	II	II	nsy	nsy
At4g02380	AtLEA5/SAG21	I	•	I	I	nsy	nsy
At3g49620	Dark inducible 11	I	•	I	nsy	nsy	nsy
At5g52750	Heavy-metal-transport superfamily	•	II	I	II	II	nsy
At1g10370	ERD9	•	•	II	I	I	nsy
At5g14700	Cinnamoyl-CoA reductase-related	•	•	II	II	II	nsy
At1g29395	AtCOR413-TM1	•	I	I	I	nsy	nsy
At2g15970	AtCOR413-PM1/Cyclophilin 19	•	•	II	II	nsy	nsy
At2g17040	Anac036	•	I	I	nsy	nsy	nsy
At2g17040	Anac036	•	I	I	nsy	nsy	nsy
At3g49530	Anac062	•	•	I	nsy	nsy	nsy
At3g25780	Allene oxide cyclase 3	•	•	I	nsy	nsy	nsy
At3g52400	AtSYP122 syntaxin	•	•	I	nsy	nsy	nsy
At5g22250	CAF1b/CCR4-associated factor 1	•	•	I	nsy	nsy	nsy
At3g44260	CAF1a/CCR4-associated factor 1	•	•	II	nsy	nsy	nsy
At5g47230	ERF5	•	•	I	nsy	•	nsy
At1g28370	ERF11	•	•	II	nsy	•	nsy
At4g37150	Methyl esterase 9	•	•	II	I	•	nsy
At4g23600	AtCORI3	•	•	I	I	nsy	nsy
At2g42540	AtCOR15A	•	•	II	II	nsy	nsy
At3g19580	AZF2	•	•	II	II	nsy	nsy
At3g50970	Dehydrin XERO2	•	•	II	II	nsy	nsy
At2g46270	G-box binding factor 3	•	•	II	II	•	nsy
At5g13200	GRAM domain-containing protein	•	•	II	II	nsy	nsy
At2g28400	Protein of unknown function	•	•	II	II	nsy	nsy
At1g60190	AtPUB19 E3 ubiquitin ligase	•	•	II	II	nsy	nsy
At1g19180	TIFY10A/JAZ1	•	•	II	II	nsy	nsy
At5g59820	ZAT12	•	•	II	II	•	nsy
At5g04340	ZAT6	•	•	II	II	nsy	nsy
At5g05300	Protein of unknown function	•	•	I	II	nsy	nsy
At4g36990	Heat shock factor protein 4	•	•	I	II	nsy	nsy
At2g32030	GNAT family protein	•	•	I	nsy	II	nsy
At2g39030	GNAT family protein	•	•	I	I	nsy	nsy
At3g63010	Gibberellin receptor GID1B	•	•	I	I	nsy	nsy
At1g65690	LEA-related/HIN1-related	•	•	I	I	nsy	nsy
At3g22600	Lipid-binding protein	•	•	I	I	nsy	nsy
At2g32100	Ovate family protein 16	•	•	I	I	nsy	nsy
At4g20110	Vacuolar sorting receptor 7	•	•	I	I	nsy	nsy
At5g54490	EF-hand containing PBP1	•	•	I	nsy	nsy	nsy
At4g39030	MATE-transporter SID1/EDS5	•	•	I	nsy	nsy	nsy
At2g43320	Putative methyltransferase	•	•	I	nsy	nsy	nsy
At3g46930	Protein kinase family protein	•	•	I	nsy	nsy	nsy
At3g09830	Protein kinase family protein	•	•	I	nsy	nsy	nsy
At2g30020 At3g27140 At4g08260	Protein phosphatase 2C	•	•	I	nsy	nsy	nsy
At4g22590	Trehalose-6-phosphate phosphatase	•	•	I	nsy	nsy	nsy
At1g66090	TIR-NBS disease resistance protein	•	•	I	nsy	•	nsy
At5g41740 At5g41750	TIR-NBS disease resistance proteins	•	•	II	nsy	nsy	nsy
At5g20230	Blue-copper binding SAG14	•	•	II	nsy	•	nsy
At1g02400	Gibberellin oxidase 6	•	•	II	nsy	nsy	nsy
At3g62260	Protein phosphatase 2C	•	•	II	nsy	nsy	nsy
At3g04640	RNA-binding glycine-rich protein	•	•	II	nsy	nsy	nsy
At4g35480	RING finger protein RHA3B	•	•	II	nsy	nsy	nsy
At1g05340	Protein of unknown function	•	•	II	nsy	•	nsy
